# Irisin Ameliorates Hypoxia/Reoxygenation-Induced Injury through Modulation of Histone Deacetylase 4

**DOI:** 10.1371/journal.pone.0166182

**Published:** 2016-11-22

**Authors:** Yu Tina Zhao, Hao Wang, Shouyan Zhang, Jianfeng Du, Shougang Zhuang, Ting C. Zhao

**Affiliations:** 1 Department of Surgery, Roger Williams Medical Center, Boston University Medical School, Boston University, Providence, RI, United States of America; 2 Department of Cardiology, Luoyang Central Hospital affiliated to Zhengzhou University, Luoyang, Henan, China; 3 Department of Medicine, Rhode Island Hospital, Brown University, Providence, RI, 02903, United States of America; University of Cincinnati College of Medicine, UNITED STATES

## Abstract

Irisin is a recently identified myokine which brings increases in energy expenditure and contributes to the beneficial effects of exercise through the browning of white adipose tissues. However, its effects in the heart remains unknown. This study sought to determine the effects of irisin on hypoxia/reoxygenation injury and its relationship with HDAC4. Wild type and stable HDAC4-overexpression cells were generated from H9c2 cardiomyoblasts. HDAC4 overexpression cells and wild type H9c2 cells were exposed to 24 hours of hypoxia followed by one hour of reoxygenation in vitro in the presence or absence of irisin (5 ng/ml). Cell cytotoxicity, apoptosis, mitochondrial respiration, and mitochondrial permeability transition pore (mPTP) were determined. Western blotting was employed to determine active-caspase 3, annexin V, and HDAC4 expression. As compared to wild type H9c2 group, HDAC4 overexpression remarkably led to a great increase in cell death as evident by the increased lactate dehydrogenase (LDH) leakage, ratio of caspase-3-positive cells as well as the upregulated levels of active-caspase 3 and annexin V shown by western blot analysis. In addition, HDAC4 overexpression also induced much severe mitochondrial dysfunction, as indicated by apoptotic mitochondria and increased mPTP. However, irisin treatment significantly attenuated all of these effects. Though irisin treatment did not influence the expression of HDAC4 at the transcriptional level, western blot analysis showed that HDAC4 protein levels decreased in a time-dependent way after administration of irisin, which is associated with the degradation of HDAC4 mediated by small ubiquitin-like modification (SUMO). Our results are the first to demonstrate that the protective effects of irisin in cardiomyoblasts exposed to hypoxia/reoxygenation might be associated with HDAC4 degradation.

## Introduction

Irisin is a recently identified proliferator-activated receptor-gamma coactivator-1α (PGCγ-1α)-dependent myokine, and is secreted by skeletal muscle and myocardium into circulation during exercise as a cleavage product of the extracellular portion of type I membrane protein fibronectin type III domain containing 5 (FNDC5) [1]. It was initially discovered as a hormone responsible for the beneficial effects of exercise through inducing the browning of white adipose tissues and increases in energy expenditure[1]. Irisin has also been demonstrated to reduce oxidative stresses and apoptosis in different models [2, 3]. Recent evidence has indicated that irisin could induce the browning of white adipose tissue, which could then be used as a therapeutic tool for metabolic disorders and cardiovascular diseases [4]. The systemic administration of irisin was protective against endothelial injury and ameliorated atherosclerosis in an apoE (-/-) diabetic mouse model, indicating that irisin could be beneficial for atherosclerotic vascular diseases in diabetes [5].

Histone acetyltransferases (HAT) and histone deacetylases (HDAC) have emerged as important mechanisms in the regulation of a variety of cellular responses [6]. HDAC inhibition’s cardioprotective effects against injury are well identified [7, 8]. Our recent observations demonstrate that HDAC inhibition enhanced myocardial repair in vivo through the stimulation of endogenous regeneration [9]. This is in line with our findings showing that HDAC inhibition facilitated embryonic stem cell differentiation into cardiac lineages and also enhanced resistance to oxidative stress [10, 11]. We have demonstrated that the specific inhibition of HDAC4 in cardiac progenitor cells promoted cardiac functional improvements in the stem cell-engrafted heart and suppressed myocardial remodeling [11].

We subjected HDAC4 to regulation by sumoylation through SUMO-1, which resulted in HDAC4 degradation [12]. More importantly, we found that infection of HDAC4 adenovirus in cardiomyocytes enhanced susceptibility to hypoxia/reoxygenation while knockdown of HDAC4 increased the resistance of myocytes to hypoxia/reoxygenation-induced injury [13]. Nevertheless, there are no current studies which determine whether irisin could generate protective effects against hypoxia and reoxygenation injury in cardiomyocytes and whether this protective effect could be related to HDAC4 signaling. In this study, we will determine 1) the effects of irisin on hypoxia/reoxygenation-induced injury in cardiomyoblasts; 2) whether the effects of irisin on hypoxia/reoxygenation injury are associated with mitochondrial function; 3) whether irisin can rescue the detrimental effects of HDAC4 over-expression in cardiomyocytes. Our results indicate that irisin produces protective effects against hypoxia/reoxygenation-induced injury in cardiomyocytes and improved the function of mitochondria, which is related to HDAC4 degradation.

## Methods and Materials

### In vitro H9c2 cardiomyoblast culture and establishment of H9c2 over-expression HDAC4 cardiomyoblasts

**Fig 1:**
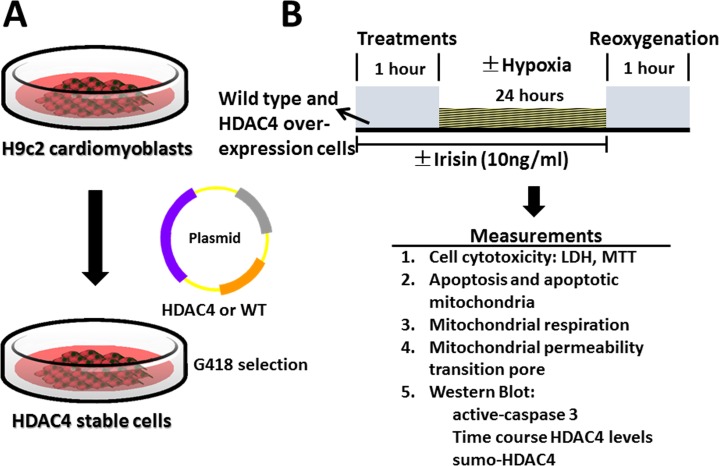
The experimental protocol. (A) Generation of HDAC4 overexpression cell. (B) Hypoxia/reoxygenation experimental protocol in H9C2 cardiomyoblasts and HDAC4 overexpression cells.

H9c2 cardiomyoblasts were purchased from American Type Culture Collection (ATCC, Manassas, VA). Cells were cultured in Dulbecco's Modified Eagle Medium (DMEM) supplemented with 10% heat-inactivated fetal bovine serum (FBS) and 1% penicillin/streptomycin at 37°C in a humidified atmosphere of 5% CO_2_. The pcDNA-3.1 wild type (WT) and pcDNA3.1-HDAC4 plasmids were generous gifts from Dr. Ronald T. Hay (University of St. Andrews, UK). To achieve stable cell lines, H9c2 cardiomyoblasts were transfected with plasmids encoding pcDNA-3.1-wild type and/or HDAC4 plasmids using Lipofectamine 2000 (Life Technologies, Grand Island, NY); these cell lines were designated as wild-type and HDAC4 cells in this study, respectively (Fig 1A). After forty-eight hours of transfection, G418 (500 μg/ml, EMD Biosciences) was added to the culture medium. Clones were selected after two weeks. Stable transfectants were maintained in regular DMEM medium containing 100 μg/ml of G418.

### Reagents and antibodies

Irisin was purchased from Cayman Chemical (Michigan, USA). The MitoCapture mitochondrial apoptosis detection kit was obtained from BioVision (Tokyo, Japan). Active-caspase 3 polyclonal rabbit antibody was obtained from Abcam (MA, USA). Primary antibodies including polyclonal rabbit β-actin and polyclonal rabbit small ubiquitin-like modifier (sumo-1) were purchased from Santa Cruz Biotechnology (Santa Cruz, CA). HDAC4 polyclonal rabbit primary antibody HDAC4 was purchased from Cell Signaling (Cell Signaling ^Tm^, Beverly, MA)). 3-[4,5-dimethylthiazol-2-yl]-2,5-diphenyltetrazolium bromide (MTT) and 4,6-Diamidino-2-phenylindole (DAPI) were obtained from Life Technologies (Grand Island, NY).

### Hypoxia/reoxygenation protocol

The hypoxia/reoxygenation protocol was the same as described previously with modifications [13, 14]. When cardiomyoblasts grew to approximately 70~80% of confluence, cells were pre-starved using DMEM supplemented with 1% FBS for 2 h, and then followed by either irisin treatment (5ng/ml) for 1 h or no irisin treatment; this procedure is shown in Fig 1B. Cells were subjected to either normoxia or hypoxia in a hypoxic chamber filled with low O_2_ gas containing 1% O_2_, 5% CO_2_ and 94% N_2_ for 24 h in the presence or absence of irisin, followed by 1 h of reoxygenation. Following H/R, cells were harvested for the examination of cell viability, death, apoptosis, mitochondrial functions and signaling pathway using western blot analysis.

### Determination of cell death and cell viability

Following H/R, injury index was assessed by measurement of LDH release in the supernatant, which is described in the previous section [15]. Following treatment as outlined in the H/R protocol, the culture medium was collected and centrifuged. Cytotoxicity was determined using a CytoTox 96® non-radioactive cytotoxicity assay kit (Promega, Madison, WI) according to manufacturer’s instructions. In addition, the cell viability assessment was conducted based on the description and the principle of reduction of 3-[4,5-dimethylthiazol-2-yl]-2,5-diphenyl tetrazolium bromide (MTT) (Sigma-Aldrich, St. Louis, MO) into blue formazan pigments in viable cells [13,15]. At the end of the experiment, the medium was removed, and the cells were washed with 1×PBS (PH 7.4. MTT (0.01 g/ml), dissolved in 1×PBS, and 500 μl of MTT buffer was added to each well. Cells were subsequently incubated for 4 h at 37°C. Cells were then washed twice with 1×PBS, and 1 ml of HCl isopropanol Triton (1% HCl in isopropanol; 0.1% Triton X-100; 50:1) was added to each well and incubated for 5 min. The suspension was then centrifuged at 16,000g for 2 min. The optical density was determined spectrophotometrically at a wavelength of 550 nm, and the values are expressed as percentages of normoxia control values.

### Immunochemical staining

Immunostaining was performed as described in detail previously [9,13]. At the end of H/R, cells were washed in 1×PBS, fixed via immersion in 4% paraformaldehyde for 15 min, and permeabilized by incubation in 1×PBS containing 0.1% Triton X-100 for 10 min at room temperature. Cells were then washed three times with 1×PBS, blocked with 1% BSA in 1×PBS for 1 h at room temperature, and incubated overnight with polyclonal anti-active caspase 3 antibody (Abcam, Cambridge, MA) at a dilution of 1:100 at 4°C. Following three washes with 1×PBS, cells were incubated with goat anti-rabbit Alexa Fluor 555 secondary antibody (Life Technologies) in 1×PBS for 1 h at room temperature. Cells were then counterstained with 4′,6-diamidino-2-phenylindole (DAPI) to visualize the nuclei. Active caspase-3-positive cells were identified using confocal laser scanning microscopy (LSM 700, Carl Zeiss). The percentage of apoptotic positive cells was determined in five randomly chosen fields and was normalized with the total number of stained nuclei.

### Western blot and immunoprecipitation

The methods and details for protein preparations and immunoblotting were carried out as described before [8]. In brief, the blots were incubated with their respective polyclonal antibodies, including active-caspase 3 polyclonal, annexin V polyclonal, HDAC4 polyclonal, and β-actin monoclonal antibodies at a diluted concentration of 1:1,000, then visualized by anti-rabbit or anti-mouse horseradish peroxidase-conjugated secondary antibody (1:2,000), and finally developed with ECL chemiluminescence detection reagent (Amersham Pharmacia Biotech). Immunoprecipitation was carried out as previously described [13]. In summary, cells were lysed in cold RIPA buffer at the end of experiments, and protein was separated by centrifugation at 4°C. The protein was incubated with the indicated primary antibody overnight (HDAC4 or IgG) at 4°C with gentle rotation. On the following day, the EZView Red Protein A affinity gel (Sigma-Aldrich, St. Louis, MO) was pre-washed with cold RIPA buffer three times; beads were added to lysate plus antibody mix and further incubated for 2 h at 4°C. Proteins were eluted, subjected to SDS–PAGE, and immunoblotted with rabbit-Sumo-1 antibody. HDAC4 input was then evaluated with anti-rabbit HDAC4 antibody as described.

### Mitochondrial membrane potential

A reduction in mitochondrial membrane potential is an early indicator of apoptosis induction [16]. Cardiomyoblast apoptosis was detected using MitoCapture mitochondrial apoptosis kit according to the protocol provided by the manufacturer [17,18]. Briefly, after the cardiomyoblasts were treated with H/R as described above, the cells were incubated in 1 ml of incubation buffer containing 1 μl of MitoCapture for 20 min at 37°C in an incubator. The fluorescent signals were measured using a confocal laser scanning microscopy (LSM 700, Carl Zeiss). The red fluorescent signals were excited at 530 nm and detected at 630 nm, and the green fluorescence was excited at 488 nm and detected at 530 nm.

### Mitochondrial permeability transition pore (mPTP)

The mPTP opening was measured by using the method described previously [17, 19]. In brief, the cells were washed with Hanks' balanced salt solution-10 mM HEPES (pH 7.2) before staining with 1 μmol/l calcein-AM (Molecular Probes) in the presence of 8 mmol/l cobalt chloride (CoCl_2_) at room temperature for 20 min in darkness. CoCl_2_ was added to quench the cytoplasmic signal so that only the fluorescence mitochondria were captured. Change in fluorescence intensity is an index of mPTP opening, and integrated optical density was obtained from three to four independent experiments.

### Real time PCR

Extractions of RNA and mRNA detection were carried out as previously described [13]. The total RNA of cells was extracted using Trizol reagent (Life Technologies, Grand Island, NY). cDNA was synthesized from 5 μg of total RNA. The reverse transcribed cDNA (5 μL) was amplified to a final volume of 50 μL by PCR under standard conditions. Real-time PCR experiments were performed on a MasterCycler RealPlex4 (Eppendorf North America) system using the qPCR Kit master mix (Kapa Biosystems, Boston, USA). The reaction condition was the following: 95°C for 2 min, then 95°C 15 sec, 60°C 20 sec, 72°C 20 sec for 40 cycles in 20 μl per reaction volume. Primer sequences for HDAC4 used in these studies are the following: Forward: 5-CTG CAA GTG GCC CCT ACA G-3, Reverse: 5-CTG CTC ATG TTG ACG CTG GA-3. GAPDH was used as the internal control: Forward: 5-ACC ACA GTC CAT GCC ATC AC-3; Reverse: 5-TCC ACC ACC CTG TTG CTG TA-3.

### Statistical analysis

All data are expressed as mean±SEM. Differences among groups were analyzed by one-way analysis of variance (ANOVA), followed by Bonferroni correction. A p<0.05 was considered to be of statistical significance.

## Results

### Irisin increased cell survival and rescued HDAC4-induced injury in cardiomyoblasts exposed to hypoxia/reoxygenation

**Fig 2:**
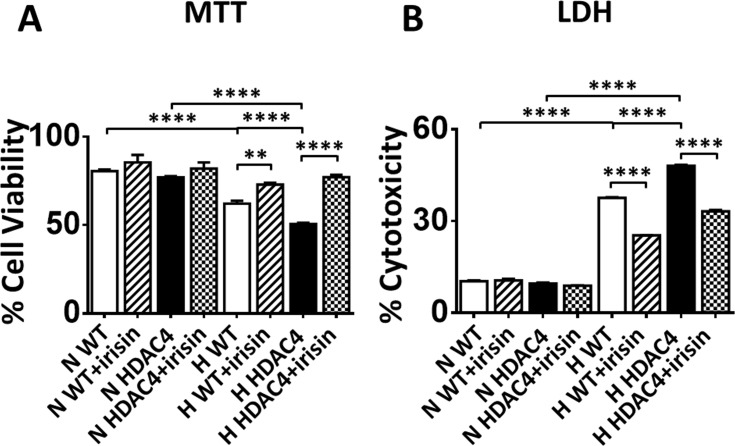
Effects of irisin on cell viability and cytotoxicity. (A) Irisin increased cell survival and rescued HDAC4-induced injury in cardiomyoblasts exposed to hypoxia/reoxygenation. (B) Irisin attenuated cytotoxicity and rescued the HDAC4-induced cell damage in hypoxia/reoxygenation. Values represent means±SEM (n = 3/group). **P<0.01, ****P<0.0001.

As compared with the normoxia condition, cardiomyocytes exposed to hypoxia/reoxygenation demonstrated cellular injury, as evident by the decrease in MTT. However, irisin treatment significantly increased the magnitude of MTT in response to hypoxia and reoxygenation, which is shown in Fig 2A. In addition, HDAC4 overexpression increased susceptibility to H/R injury compared with wild type control in Fig 2A. After H/R, H9c2 wild-cell cell viability was decreased from 88.67±0.88% in normoxia to 62.22±1.53% in H/R (p<0.0001). However, as compared to wild type cells, HDAC4 over-expression resulted in a further decrease in cell survival rate to 50.65±0.65%. Notably, the HDAC4 over-expression induced decrease in cardiomyoblast viabilities exposed to H/R were prevented with treatment of irisin. Cell viability increased from 50.65±0.65% to 77.22±1.36% with irisin treatment, suggesting that HDAC4 overexpression augmented H/R injury, but could be attenuated by neutralized irisin. However, the lower dose of irisin treatment (1ng/ml) did not elicit significant protective effects in this model (data not shown).

### Irisin attenuated cytotoxicity and rescued the HDAC4-induced cell damage in hypoxia/reoxygenation

To examine the effects of irisin on cytotoxicity, cell death by necrosis was examined by measuring the release of cytosolic LDH enzyme [20, 21]. We carried out these measurements after subjecting cells to 24h of hypoxia, followed by 1h of normoxia, as presented in Fig 1B. As shown in Fig 2B, LDH release increased from 10.31±0.33% to 37.57±0.21% in response to hypoxia/reoxygenation (p<0.0001). The magnitude of LDH release was significantly increased in the HDAC4 overexpression group. Likewise, irisin treatment remarkably prevented HDAC4-induced cell deaths, as evident by the great reduction of LDH leakage from 48.05±0.0.29% in the HDAC4 overexpression group to 33.15±0.48% following irisin treatment. These findings indicate that the augmented HDAC4 overexpression increased the susceptibility of cells to H/R, which was mitigated by irisin.

### Irisin suppressed HDAC4 induced cell apoptosis after H/R

**Fig 3:**
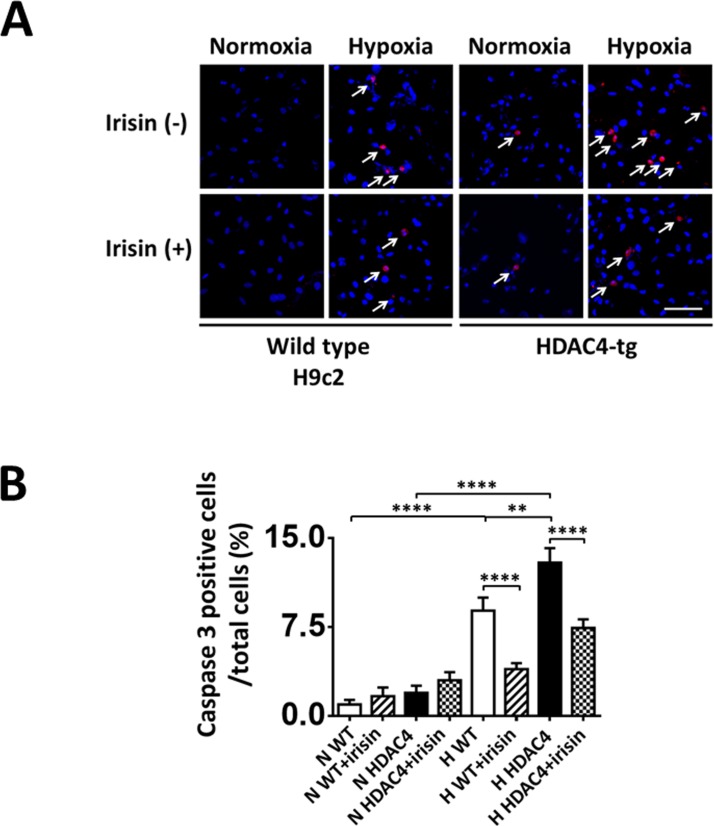
Irisin treatment reduced active caspase-3-positive nuclei in cardiomyoblasts exposed to H/R. (A) Representative images showing the apoptotic H9c2 cardiomyoblasts: active caspase-3-positive nuclei in red (white arrows); nuclei were stained in blue (DAPI). (B) Quantification of active caspase-3-positive nuclei between groups. Values represent means±SE (n = 3/group). ***P<0.001, ****P<0.0001. Scale bar: 100μm.

**Fig 4:**
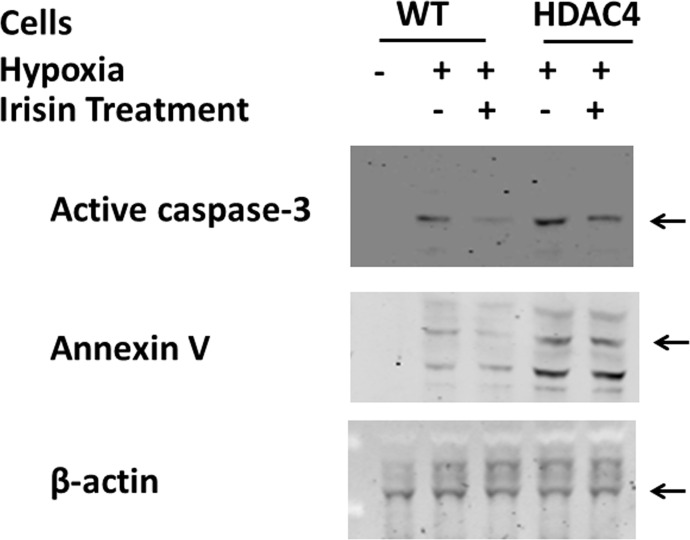
Irisin suppressed HDAC4 induced cell apoptosis after H/R. Active-caspase 3 and annexin V signals were significantly reduced by irisin treatment. Irisin mitigated the increase in both active caspase 3 and annexin V in HDAC4 overexpression group.

As shown in Fig 3A and 3B, active caspase 3 was highly exhibited in H9c2 cardiomyoblasts exposed to hypoxia/reoxygenation injury, as results show an increase from 0.96±0.39% in normoxia to 8.88±1.10% in response to hypoxia/reoxygenation. However, the treatment of irisin decreased the number of active caspase 3 positive cells. Additionally, as compared to wild type cells, HDAC4 over-expression further enhanced the rate of active-caspase 3 signals under the condition of hypoxia and reoxygenation which was elevated from 8.88±1.10% in wild type to 12.93±1.22% in the HDAC4-over-expression group. There were no differences in positive active caspase 3 cell number between wild type and HDAC over-expression group under the normoxia condition, indicating that HDAC4 over-expression under normoxia conditions may not be sufficient enough to elicit an apoptotic pathway in this model. Strikingly, irisin treatment significantly reduced the percentage of apoptotic cells in the HDAC4 overexpression model, suggesting that irisin attenuated apoptosis in cardiomyoblasts exposed to hypoxia and reoxygenation and also mitigated HDAC4-induced apoptosis. In accordance with this observation, as displayed in Fig 4, Western blot analysis showed that the active-caspase 3 and annexin V signals were significantly reduced by irisin treatment, and irisin mitigated the increases in both active caspase 3 and annexin V levels in HDAC4 overexpression group.

### Irisin protects against hypoxia/reoxygenation-induced mitochondrial damage

**Fig 5:**
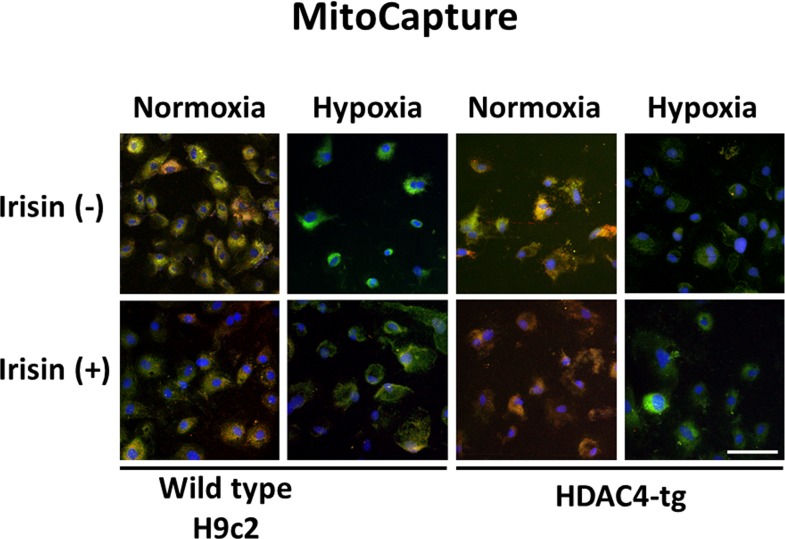
The effect of irisin on H/R induced MMP reduction in H9c2 cells. Cardiomyoblast mitochondrial damage was assessed by examining mitochondrial membrane depolarization. The MitoCapture dye accumulates in the mitochondria under normoxia to emit a red signal. In apoptotic cells, the MitoCapture diffuses into the cytoplasm and emits a green signal. Exposing H9c2 cells to H/R caused a significant decrease in the ratio of red to green fluorescence intensity, which is a sign of the early stages of cell apoptosis. Apoptosis was more severe in the HDAC4 group. However, irisin treatment improved the H/R-led MTP loss significantly in both WT cells and HDAC4 over-expression cells. The bar represents 100 μm.

Modification of the mitochondrial membrane potential (MMP) is an early event in the induction of apoptosis. To assess the state of MMP, a cationic dye in living cells, MitoCapture (Bio Vison), was used. This dye accumulates in mitochondria when the mitochondrial function is intact and emits a red signal in cells while the apoptotic mitochondria emits a green signal. As shown in Fig 5 and S1 Fig, as compared to the normoxia condition, cardiomyoblasts exposed to hypoxia/reoxygenation lost the red fluorescent signals, and this effect was prevented by treatment of irisin. Furthermore, HDAC4 over-expression resulted in a further suppression of fluorescent signals as compared to the wild type group. However, the HDAC overexpression elicited MMP loss in hypoxia/reoxygenation was mitigated by irisin treatment.

### Irisin inhibited the mPTP opening

**Fig 6:**
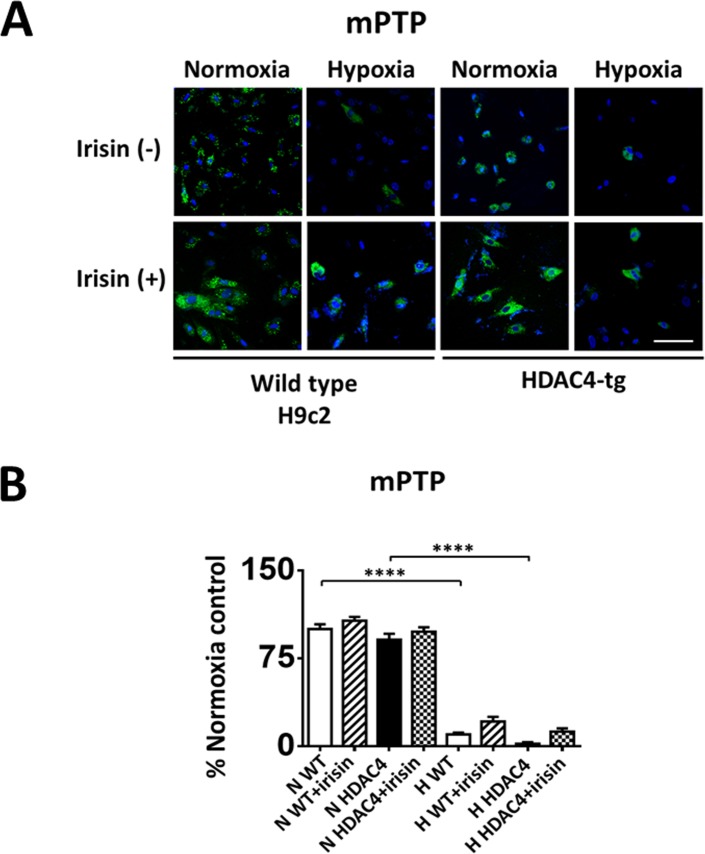
Effects of irisin on mitochondrial permeability transition pore (mPTP) opening in cardiomyoblasts exposed to hypoxia/reoxygenation. (A) Representative images of mPTP staining. The onset of mPTP is demonstrated by loss of green fluorescence signal from mitochondria. Detailed methods for measurement of mPTP were described in materials and methods. Scale bar:100 μm. (B) Quantitation analysis of mPTP in H9c2 cardiomyoblasts exposed to hypoxia/reoxygenation. Our analysis showed that irisin treatment rescued the HDAC4 induced-increase in mPTP opening. The results represent 3–4 independent experiments counting 150–200 cells per condition. Values represent means±SE (n = 3-4/group). ****P<0.0001.

The mitochondrial permeability transition pore (mPTP) plays an essential role in the pathogenesis of myocardial ischemia/reperfusion injury [22, 23]. Inhibition of the mPTP opening at the early reperfusion stage was shown to protect the heart from reperfusion [24, 25]. As shown in Fig 6A, H9c2 cells exposed to hypoxia/reoxygenation injury demonstrated a significant loss in mitochondrial green fluorescence signals as compared to normoxia. Furthermore, HDAC4 over-expression resulted in a trend of further reduction in mitochondrial green fluorescent signaling in cells, which was confirmed with quantitative analysis afterwards (Fig 6B). However, treatment with irisin rescued the HDAC4 induced increase in mPTP opening.

### Irisin promoted HDAC4 reduction and sumoylation

**Fig 7:**
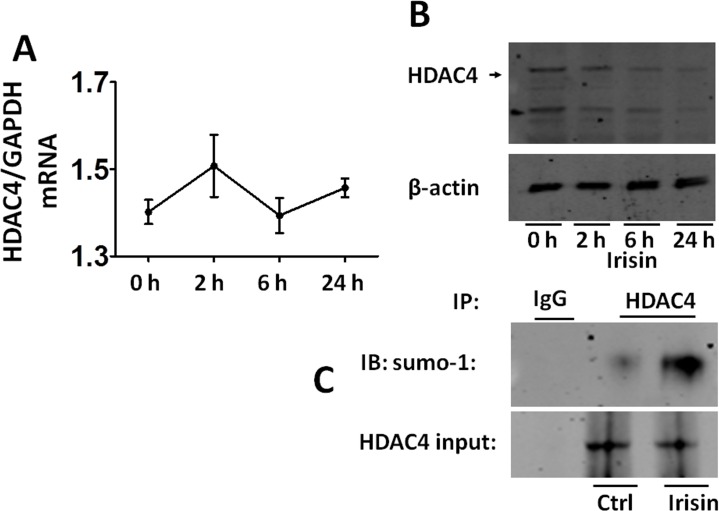
Effect of irisin on HDAC4 expression in transcriptional and protein levels. (A) Quantitative PCR results of HDAC4. It showed that irisin did not influence HDAC4 mRNA. (B) Western blot analysis of HDAC4. Irisin treatment resulted in a time-dependent decrease in HDAC4 level. The results represent 3–4 independent experiments. (C) Immunoprecipitation showing that irisin induced HDAC4 sumoylation in H9c2 cardiomyoblasts exposed to H/R. These results represent 3–4 independent experiments. IgG: Immunoglobulin G. IP: immunoprecipitation.

To further determine the relationship between irisin and HDAC4, we analyzed HDAC4 transcription protein levels and its sumoylation. As shown in Fig 7A, the real-time PCR analysis showed that treatment with irisin did not result in changes in HDAC4 mRNA. However, treatment with irisin exhibited a time-dependent decrease in HDAC4 (Fig 7B). Since we have recently demonstrated that HDAC4 sumoylation resulted in HDAC4 degradation, we performed an immunoprecipitation assay to assess if there exists an increase in HDAC4 sumoylation following irisin treatment in H9c2 cardiomyoblasts. As shown in Fig 7C, irisin treatment led to a significant increase in HDAC4 sumoylation, suggesting that irisin promoted HDAC4 sumoylation. In addition, HDAC4 sumoylation displayed a time-dependent course, which indicates that sumoylation increased following irisin treatment (**S2 Fig**). However, in this observation, it remains unknown whether the magnitude of HDAC4 sumoylation is associated with cell survival rates.

## Discussion

### Salient findings and perspectives

This study demonstrates that irisin generates protective effects in cardiomyocytes exposed to hypoxia/reoxygenation. The protective effects of irisin were also associated with the attenuation of myocardial apoptosis as well as the suppression of mitochondrial apoptosis and inhibition of mitochondrial PTP. Furthermore, irisin elicits the sumoylation of HDAC4 and led to a time-dependent HDAC4 degradation. HDAC4 overexpression increased the susceptibility of cardiomyoblasts exposed to hypoxia/reoxygenation, but these effects were attenuated by irisin treatment. Taken together, these results indicate that irisin produced a protective effect against hypoxia/reoxygenation-induced injury in association with inducing the improvement of mitochondrial function and reduction of apoptosis. Treatment with irisin rescued cardiomyocytes from the detrimental effects of HDAC4 overexpression under hypoxia/reoxygenation.

Recent evidence has well addressed the physiological function of irisin in modulating body metabolism and thermogenesis [1, 26, 27]. The major functions of irisin on metabolic syndrome include not only the driving of the browning of white adipose tissue, which then increases energy expenditure, but also include the suppression of inflammation and oxidative stress [2, 5, 28, 29]. In these studies, we found that treatment with irisin effectively attenuated cell death and increased the survival rate of cardiomyocytes exposed to hypoxia and reoxygenation, therefore establishing that irisin serves as a novel approach to trigger protective effects. In line with our observations, we have also recently found that administration of irisin improved myocardial function recovery and decreased myocardial infarct size (unpublished data). On the other hand, although we observed that irisin reduced cell death and apoptosis under hypoxia, we did not evaluate the effect of irisin on cell necrosis, which is a limitation and holds merit for the future investigations.

We have previously demonstrated that GLP-1 and GLP-1R stimulation produced a peptide, which is critical to attenuate myocardial injury and to suppress the development of diabetic cardiomyopathy. The protective effects were closely associated with the improvement of mitochondrial respiration and inhibition of mitochondrial apoptosis [17, 30]. Our study here showed that irisin improved mitochondrial function by preventing the loss of mitochondrial membrane potential and suppressing the mitochondrial PTP opening, which could be critical for irisin’s protective effects. Although both GLP-1R and irisin are considered to reduce metabolic disorder, it is not clear whether they generated these protective effects in cardiomyoblasts through distinctive pathways. This inquiry holds merit for future investigation.

HDAC inhibitors were extensively tested in many disease models to achieve their therapeutic effects. We and others have demonstrated that HDAC inhibitors triggered myocardial protection against cellular and ischemic injuries [7, 10, 11, 31]. Likewise, HDAC inhibitors have produced anti-hypertrophic effects in the heart and other disease models [32–35]. More importantly, HDAC4, an isoform of HDACs in the heart, was demonstrated to be critical in the regulation of cellular injury and survival. This suggests that the targeting of HDAC4 could serve as an important model to understand the cellular mechanism(s) of HDAC4 in the development of pathological disorders. Our recent observations indicate that HDAC4 sumoylation elicited the degradation of HDAC4 following the pharmacologic inhibition of HDAC activity [13], which is crucial for the development of the cellular protective pathway. However, it is not very clear whether irisin treatment responds to HDAC4 overexpression in the presence of hypoxia, which promotes us to identify a relationship between irisin and HDAC4 in this observation. In our previous studies, the H9c2 cardiomyoblast is a well-established model used to examine cellular injury under hypoxia, which provides us the basis to utilize this model in these studies. In this study, our observation shows that irisin treatment led to the subsequent degradation of HDAC4 without changes in transcriptional levels. Furthermore, we detected an association of HDAC4 and SUMO-1 and irisin treatment induced sumoylation of HDAC4, suggesting that irisin caused the degradation of HDAC4. This is likely to be through the regulation of sumoylation. It is also likely that irisin stimulates HDAC4 ubiquitination, which was directly modulated by its sumoylation [13]. Additionally, irisin treatment stimulated greater HDAC4 accumulation in nuclei, so it is not clear whether the distribution of HDAC4 was associated with the protective effects elicited by irisin. Our previous works indicated that HDAC4 was up-regulated in response to oxidant stress, and genetic inhibition of HDAC4 promoted myocardial regeneration [10], implying that HDAC4 may function as a critical HDAC isoform attributable to cardioprotection and repair. HDAC4 overexpression increased hypoxic-induced cell damage in cardiomyocytes NMVM, addressing the importance of HDAC4 in determining cell survival in response to stress. By using genetic HDAC4 overexpression cardiomyoblasts, we demonstrated that over-expression of HDAC4 exacerbated cell death and attenuated cell survival rate in association with the depressed mitochondrial function in response to hypoxia/reoxygenation. Notably, pretreatment with irisin mitigated the magnitude of HDAC4 over-expression induced cellular damages, indicating that irisin could rescue the detrimental effects of HDAC4, which is likely to result from the reduction of HDAC4 proteins following irisin treatment.

In conclusion, our study demonstrated that irisin prevents cell death, increases cell survivals, and reduces apoptosis in cardiomyocytes exposed to H/R. The protective effects of irisin are closely associated with the inhibition of mitochondrial PTP and prevents the loss of mitochondrial membrane potential. Irisin elicits time-dependent reductions in HDAC4 and increases in HDAC4 sumoylation. Furthermore, overexpression of HDAC4 enhanced cell death and attenuated cell survival rate, which is associated with the disturbance of mitochondrial function. The HDAC4 overexpression-enhanced H/R injury was rescued by pretreatment of irisin. Our studies provide new insight into the understanding of the functional role of irisin/HDAC4 working module and hold promise in developing irisin as a new therapeutic strategy.

## Supporting Information

S1 Fig The effects of irisin on H/R induced MMP reduction in H9c2 cells. Quantification of the emitted fluorescent signal was achieved by calculating the average value of intensity within marked edges. They were corrected by calculating the mean intensity of 30 cell-free fields and the results are shown as means±SEM. *P<0.05, ***P<0.0001.
(TIF)

S2 Fig Time-course of HDAC4 sumoylation following irisin treatment. The detailed methods for immunoprecipitation and immunoblotting were described in the methods of main manuscript. Each blot represents three individual experiments.
(TIF)

S1 File Measurement of mitochondrial membrane potential. (DOCX)
