# Supplementary figures and images for: Irisin Ameliorates Hypoxia/Reoxygenation-Induced Injury through Modulation of Histone Deacetylase 4
Source: PLoS One. 2016 Nov 22;11(11):e0166182. doi: 10.1371/journal.pone.0166182 (PMC5119735; doi:10.1371/journal.pone.0166182)

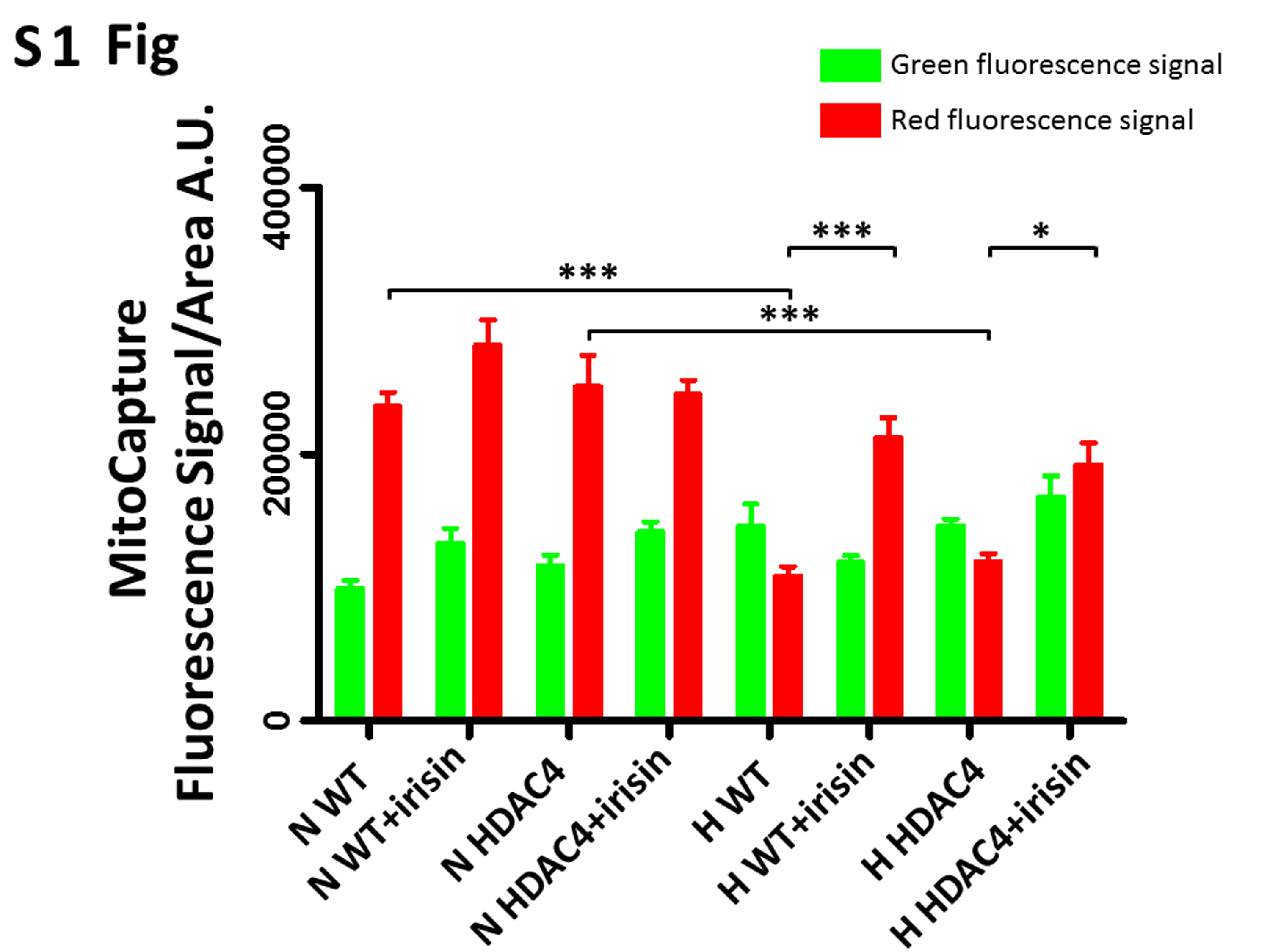

Supplement: S1 Fig — Quantification of the emitted fluorescent signal was achieved by calculating the average value of intensity within marked edges. They were corrected by calculating the mean intensity of 30 cell-free fields and the results are shown as means±SEM. *P<0.05, ***P<0.0001. (TIF) [file pone.0166182.s001.tif]

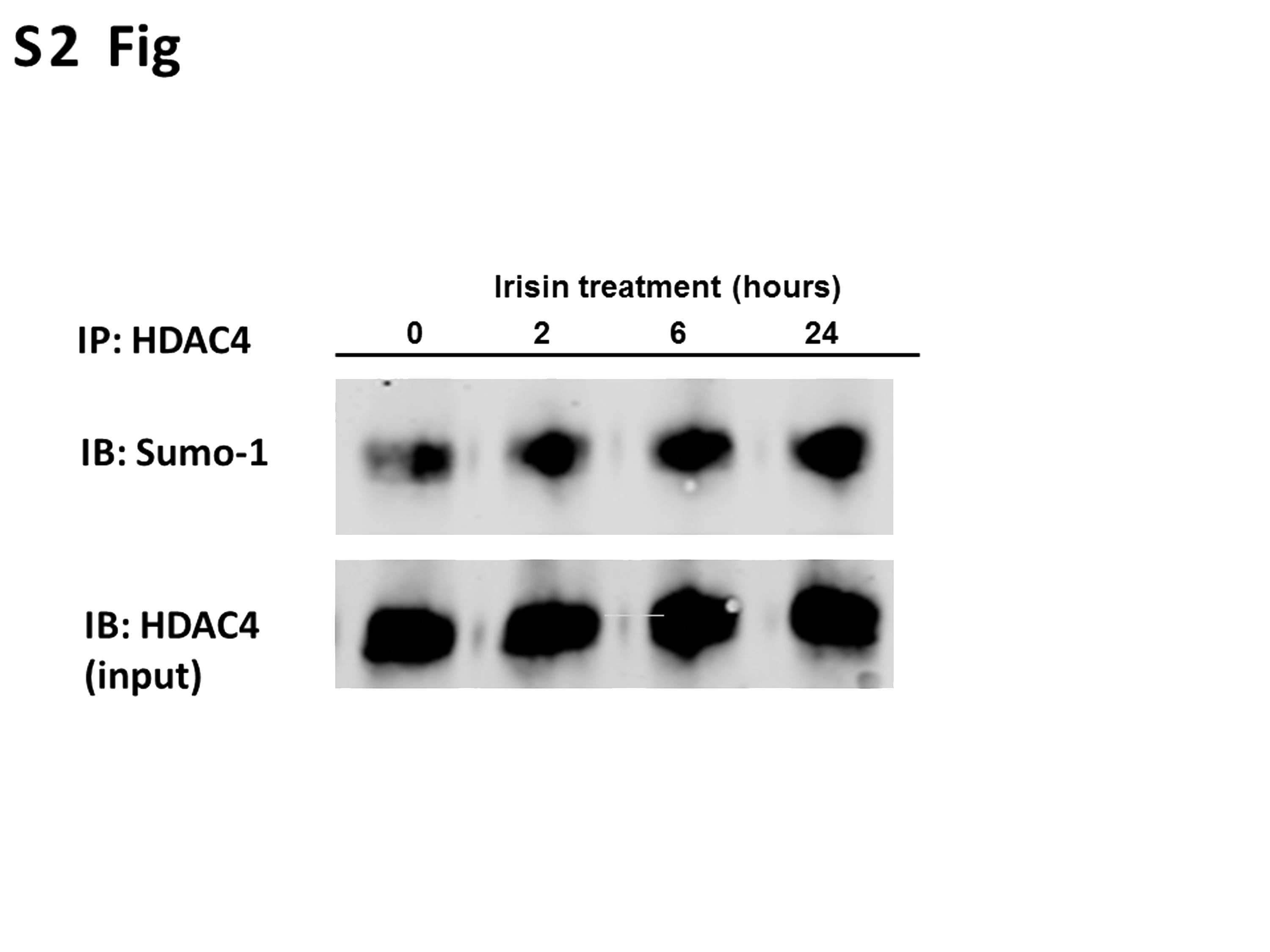

Supplement: S2 Fig — The detailed methods for immunoprecipitation and immunoblotting were described in the methods of main manuscript. Each blot represents three individual experiments. (TIF) [file pone.0166182.s002.tif]
