# Supplementary material for: Irisin Ameliorates Hypoxia/Reoxygenation-Induced Injury through Modulation of Histone Deacetylase 4
Source: PLoS One. 2016 Nov 22;11(11):e0166182. doi: 10.1371/journal.pone.0166182 (PMC5119735; doi:10.1371/journal.pone.0166182)
Supplement: S1 File — (DOCX) [file pone.0166182.s003.docx]

**Supplemental Data**

**Measurement of mitochondrial membrane potential**. Apoptosis of cardiomyoblasts was detected using MitoCapture Mitochondrial Apoptosis Kit according to protocols provided by the manufacturer, which was provided in the content of the manuscript. Briefly, after the cardiomyoblasts were treated with H/R as described above, the cells were incubated in 1 ml of incubation buffer containing 1ul of MitoCapture for 20 min at 37°C in an incubator. The fluorescent signals were measured using a confocal laser scanning microscopy (LSM 700, Carl Zeiss). The red fluorescent signals were excited at 530 nm and detected at 630 nm, and the green fluorescence signals were excited at 488 nm and detected at 530 nm. Quantification of the emitted fluorescent signal was achieved by calculating the average intensity value within marked edges, including a single cell, and quantified in arbitrary units. A total of 30 cells were analyzed for each condition, and the results are shown as means±SEM. Corrections were made to minimize background influences by calculating the mean intensity in 30 cell-free fields in each section.
